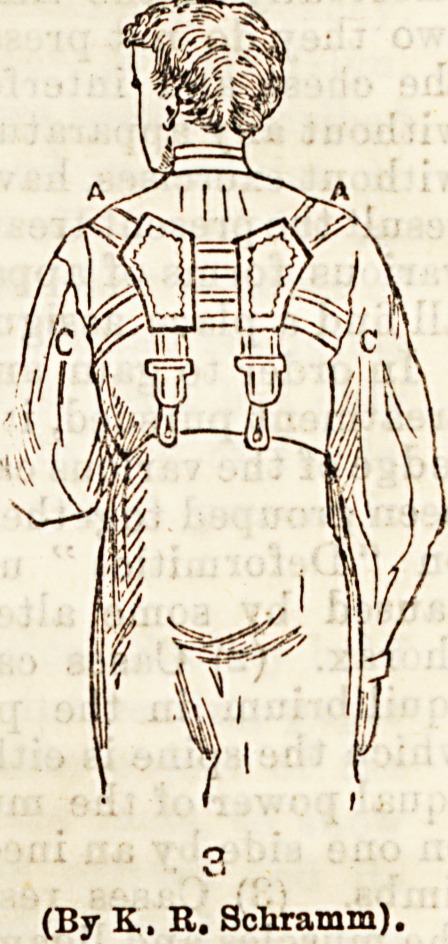# Treatment of the Lateral Curvature of the Spine

**Published:** 1893-07-29

**Authors:** 


### The Hospital Clinic.

[*The Editor will be glad to receive offers of co-operation and contributions from members of the profession. All letters should be addressed to* The Editor, The Lodge, Porchester Square, London, W.]

### ROYAL ORTHOPÆDIC HOSPITAL.

### Treatment of the Lateral Curvature of the Spine.

With regard to this disease the prevalent opinion at the Royal Orthopædic Hospital is that though some of the cases are curable, yet in many it is impossible to hope for more than the cessation of the morbid process. Under these circumstances it is natural that very great care is taken to detect the commencement of the trouble and prevent its further advance.

In the early stages there is no actual deformity of the structures forming the spine, and it is generally possible for the patient to straighten it by an effort to hold himself upright. Later on, however, this becomes impossible, no active effort can completely straighten the spine, nor can it be effected by applied force. By this time there has taken place distortion of the vertebræ or of the intervertebral discs, or these structures and the ribs to which they are attached have rotated and become fixed in the bad position.

All forms of apparatus and all sorts of exercises have been tried at the Royal Orthopædic Hospital at some time or other. Plaster of paris jackets were given up because they were found to be dirty, cumbrous, and inefficient. Sayre's poro-plastic jackets have been discarded, because it has been found that although they may press on the deformed parts pretty effectually at the time of moulding, yet after a day or two they do not press at all. Further they constrict the chest and interfere with respiration. Exercises without any apparatus, and apparatus and recumbency without exercises, have in turn been tried, and as the result the present treatment has been fixed upon in which various forms of apparatus, recumbency, and exercises all find a place assigned to them in the process of cure.

In order to gain an intelligent apprehension of the treatment pursued, it is necessary to have some knowledge of the various causes of deformity. The cases have been grouped together by Mr. Henry Baker in his book on "Deformities" under three heads. (1) "Cases caused by some alteration in the contents of the thorax. (2) Cases caused mechanically by the loss of equilibrium in the parts outside the chest walls, by which the spine is either dragged to one side by the unequal power of the muscles on the two sides, or tilted on one side by an inequality in the length of the lower limbs. (3) Cases resulting from simple weakness of the muscles and ligaments. This condition allows the spine to deviate from the straight line, and the patient not being able to exert sufficient muscular power to overcome the deformity, it becomes permanent."

In deciding on the line of treatment in any particular case it is necessary to bear these points in mind. For instance, if the leg on one side be shorter than on the other, it is obvious that there will be curvature of the spine produced, since the pelvis will be tilted, and therefore, since the spine in its lower part will be bent in one direction, it is necessary, in order to maintain the body upright, that it be bent in its upper part in the other. It is obvious that the rational treatment of this case is to order a boot with a cork sole, and so make the legs of the same length. If the case is seen early enough this is all that is required. Similarly, lateral curvature of the spine may be due to flat foot or to knock knees. These cases, of course, are treated by appropriate means—in the one case, boots and sole plates are ordered to support the sunken arch; in the other, a suitable iron is applied to support and correct the deformity of the leg.

With regard to another great class of cases, that spoken of above as resulting from some change in the organs contained inside the thorax, it is obvious that it is hopeless to expect a cure. Suppose, for instance, that in consequence of an empyema one side of the chest has fallen in, and as a natural result the spine has become bent and twisted, it is impossible to restore the chest to its natural condition, and until that is done it is not possible to straighten the curvature of the spine. These cases are treated differently according to the condition in which they come under observation. If the case is of old standing, and has not changed for a long time, no treatment is necessary. If, on the other hand the curvature is still progressing, it is desirable to order an instrument that will in some degree support the spine, and perhaps restrain the deformity within moderate limits. In ordinary cases the instrument shortly to be described is ordered, but sometimes in slight cases it is considered sufficient to give a pair of well-fitting stays, with crutches fixed to their sides, so as to support the shoulders and hold them back. In certain of these cases if the patient be weakly, even if the disease has already come to a standstill, an instrument is considered desirable to remove the sense of weakness of which some of these patients complain.

Excluding the cases already referred to, in which there is some definite and obvious reason for the curvature of the spine, there is a much larger class in which the cause is not thus obvious. These cases are mainly met with between the ages of fourteen and twenty-five. In the greater number of cases there is no obvious sign of serious ill-health; of course there may be such appearance, but it is not distinctive of this condition. Most of the patients are engaged indoors, either in sedentary occupations or in the employment as shop girls, where they have to spend long periods on their feet. In some of these cases it appears as if the disease had been started by some faulty habit of the patient, such as standing on one side, or sitting with the body twisted; but even in these cases it is probable that there was something wrong before, and that the tendency to droop over on one side was the curvature that followed it, the result of weakness of the spinal muscles. The general course of these cases is that at first the deformity is slight, and disappears when the patient holds herself up, or lies in bed, but that later it becomes permanent. As a rule the deformity goes on increasing until the patient attains her full development, and then ceases to advance, but it may not stop until it has advanced to a very serious extent, and the health has been permanently damaged.

The treatment made use of in these cases is intended to carry out the following principles: (1) Improvement of the general health and correction of faulty habits. (2) Strengthening the muscles of the spine. (3) Removal of the weight of the head and trunk as much as possible from the spine. (4) The application of an instrument which by appropriate pressure on the prominent parts of the curvature may push the spine into its right shape, or may at any rate prevent the deformity from getting worse.

The general health is improved by such measures as each case demands. Tonics are given if necessary. Fresh air is recommended, and a plentiful supply of good food.

The attempt is made to strengthen the muscles of the back by means of friction with some liniment, in addition to which various extension exercises are enjoined. If the patient is strong enough swinging by the hands is recommended, and skipping, but especially skipping backwards. It is impossible to fully describe the exercises that are made use of. The intention of them one and all is to systematically bring one after another of the muscles into use, and so promote their growth. If patients are able to bear it, a cold sponge bath every morning is found of great advantage.

Apparatus for correction of lateral curvature of the spine. (K. R. Schramm).

Recumbency for several hours a day is ordered in all cases. This is considered as of great importance. It is well known that even in the normal spine there is a certain increase in height gained during the night while at rest. This is, of course, caused by the elasticity of the intervertebral discs enabling them to throw off the effects of the pressure to which they have been subjected during the day. It is hoped that in the deformed state this same process may take place, and the intervertebral discs regaining their shape may restore the spinal column to its natural condition.

The principle of removing the weight of the upper parts of the body from the spine is only very imperfectly fulfilled by means of instruments. If they are tight enough to hold the body up, they are uncomfortable, and cause distress that more than counterbalances any advantage that could be gained by their use. For this part of the treatment then we have to rely mainly on the recumbency already spoken of. It is true that the instrument to be described does partially support the body, but the main actual result of the crutches that are supposed to discharge this duty is to hold the shoulders well back. This is a most important effect, for by expanding the front of the chest they aid the breathing and indirectly improve the health.

Several instruments for lateral curvature are used by the surgeons at the Royal Orthopædic Hospital. One of these is shown in the diagram (*b*). It consists of a firm, close-fitting steel belt round the pelvis, from each side of which springs an upright, which bears on its upper end a crutch. This projects forwards from the armpit, and turns upwards in front of the shoulder. To the uprights are attached a broad belt, which laces up the front and holds the instrument in position. From the centre of the back rise two steel rods. These are attached to the pelvic belt by a double rack and pinion joint, so that each has the power of moving in an antero-posterior direction, and also in a plane at right angles to this. Each of these rods carries a plate on its upper end, which is made of iron covered with leather, and is moulded so as to fit the curve at the place that it is intended to exert its pressure.

On looking at the diagram of lateral curvature of the spine, it will be seen that there are two curves. These are generally distinguished as primary and secondary. It is on the parts of the back corresponding to these that the pressure is applied, a large plate being usually placed over the more prominent and a smaller over the other. In some rare cases there is only one curve. Then, of course, only one plate is used. It is difficult, however, to get it to act efficiently, since there is no counter pressure for it to act against.

Whatever instrument is used, it is first made exactly to fit, so that the pressure acts in the direction most likely to procure a good result. The direction and points of pressure are sometimes determined by grasping the back with the hands and trying to press the spine into a good position. The plates are then applied as this experiment indicates. The instrument sometimes causes a little trouble at first, and needs a good deal of attention until the back becomes accustomed to the pressure, and it is found what amount can be borne. If the skin becomes tender it is bathed with spirit, and the pressure is relaxed for a day or two.

The instrument is not worn at night, or while the patient is lying down, since at those times there is no tendency for the curvature to increase.

In many cases, although the curvature has not been completely cured the instrument may be left off after a few years as the back becomes stronger, if it is evident that the deformity is not increasing.

*Anterior Curvature of the Spine.*—This deformity, more commonly known as lordosis, is most commonly the result of some contraction about the hip-joint, whereby it is impossible to get the foot to the ground without bending the back. Its treatment depends on that of the trouble that causes it, and is too large a subject to be gone into here.

*Posterior Curvature of the Spine.* — This is generally a result of debility. It is met with especially in ricketty infants, and in weakly young people who have out grown their strength. If it is caused by rickets it must be treated by the ordinary dietetic measures. If in girls or boys it is to be treated by exercise in the open air, drilling, good food, and by other methods that improve the general health. Some cases gain a good deal of advantage from the apparatus shown in the diagram. This consists essentially of a band round each shoulder with a piece of rubber connecting the two and drawing the shoulders back.

(By K. R. Schramm).

## Figures and Tables

**Figure f1:**
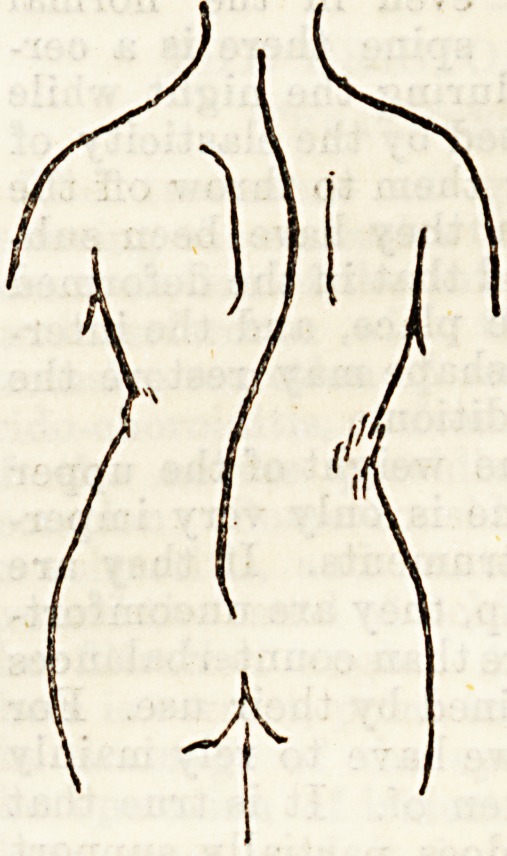


**Figure f2:**
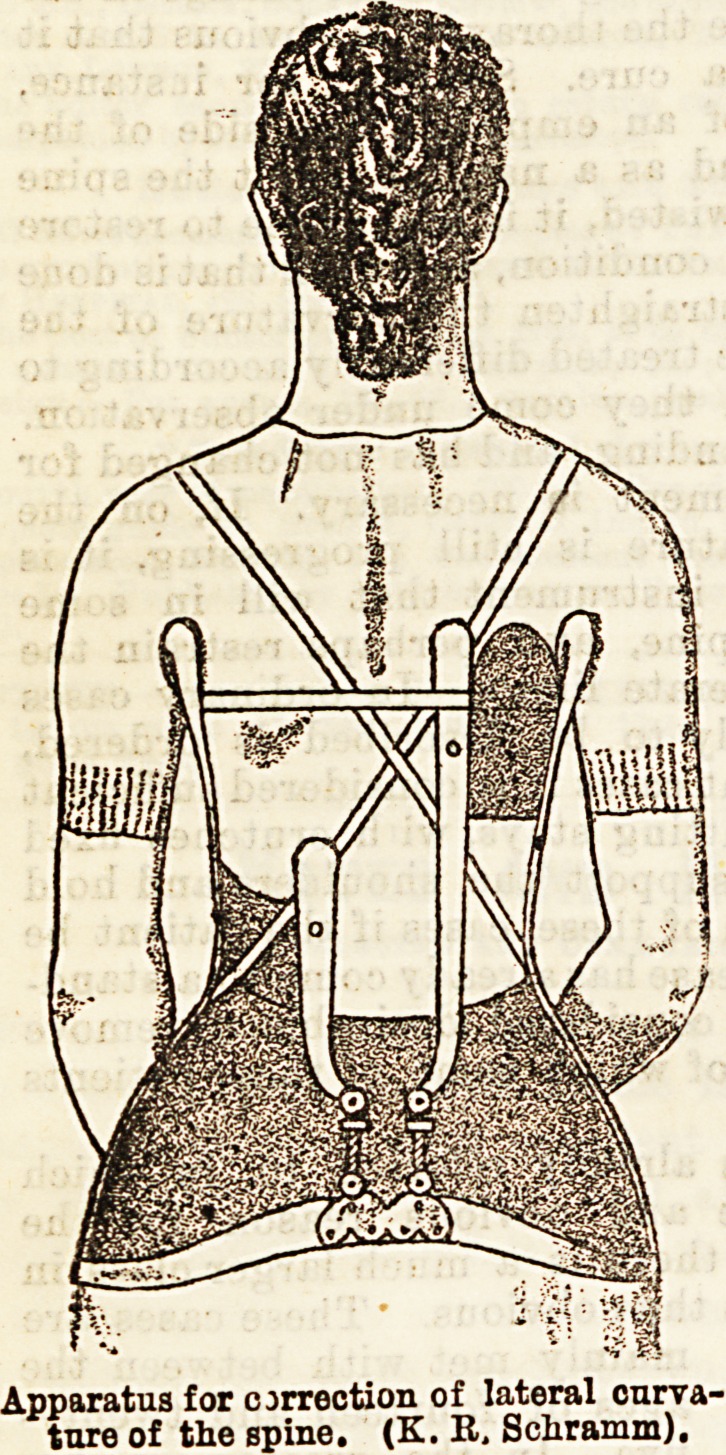


**Figure f3:**